# Chemotherapeutic potentials of the stem bark of *Balanite aegyptiaca* (L.) Delile: an antiangiogenic, antitumor and antioxidant agent

**DOI:** 10.1186/s12906-016-1369-5

**Published:** 2016-10-19

**Authors:** Loiy E. Ahmed Hassan, Saad S. Dahham, Sultan Ayesh Mohammed Saghir, Abdelhafeez M. A. Mohammed, Nagla M. Eltayeb, Amin Malik Shah Abdul Majid, Aman Shah Abdul Majid

**Affiliations:** 1 EMAN Research and Testing Laboratory, School of Pharmaceutical Sciences, Universiti Sains Malaysia, 11800 USM, Penang, Malaysia; 2 Department of Chemistry, Alzaiem Alazhari University, P.O Box 1432, Khartoum North, 13311 Sudan; 3 Department of Pharmaceutical Chemistry, School of Pharmaceutical Sciences, 11800, Universiti Sains Malaysia, Penang, Malaysia; 4 ACRF Department of Cancer Biology and Therapeutics, The John Curtin School of Medical Research Australian National University, Canberra, Malaysia; 5 Department of Pharmacology, Quest International University, Perak, Malaysia; 6 Department of Botany, Faculty of Science & Technology, Omdurman Islamic University, P.O. Box 383, Omdurman, Sudan

**Keywords:** *Balanite aegyptiaca*, Cytotoxicity, Antiangiogenic, In vivo, Antitumor, Nude mice

## Abstract

**Background:**

*Balanite aegyptiaca* (L.) Delile, is a plant with extensive medicinal properties. Its stem bark is traditionally known for its spasmolytic and antiepileptic properties and used to treat yellow fever, jaundice and syphilis. Angiogenesis (sprouting of new blood vessels) is crucial for tumor growth and metastasis. The goal of this study is investigate the antiangiogenic, cytotoxicity and antioxidant activity as well as antitumor in vivo properties of *B. aegyptiaca* stem bark extracts.

**Method:**

The dried powder of stem bark was extracted sequentially with *n*-hexane, chloroform, methanol and water. Rat aorta ring assay (RARA) was used as a platform to screen for antiangiogenic affect. The most active extract was subjected to further confirmatory antiangiogenic tests i.e. cell migration, tube formation and VEGF inhibition and finally evaluated for its in vivo antitumor efficacy in nude mice. The cytotoxicity of extracts on four cancer cell lines (HCT-116, K562, U937 and MCF-7) and one normal cells line (HUVEC) was evaluated. To assess the antioxidant activity screening, four methods were used, (DPPH•) and ABTS radical scavenging activity, as well as total flavonoids and phenolic contents.

**Results:**

Methanol extract of *B. aegyptiaca* stem bark (MBA) showed the highest antiangiogenic, antioxidant and anticancer properties. It was found selectively cytotoxic to leukemia cell lines as well as breast cancer cell line MCF-7. (MBA) thus exhibited antiangiogenic in *ex-vivo* rat aorta ring model; it was found to excel its antiangiogenic effect *via* inhibition of the key growth factor (VEGF) as well as to halt HUVEC cell migration and tube formation, furthermore animals bearing colon cancer treated with (MBA) showed significant reduction in tumor growth.

**Conclusion:**

Different extracts of *B. aegyptiaca* stem bark showed various anticancer and antiangiogenic properties. MBA demonstrated potent antiangiogenic, antioxidant and antitumor in vivo. The outcome of this study suggests the potential of stem bark of the *B. aegyptiaca* for developing chemotherapeutic agent against solid tumor as well as leukemia.

## Background

*Balanite aegyptiaca* is an evergreen xerophytic tree (known as desert date) of huge medicinal importance in Sudanese traditional medical practice. It belongs to the family *Balanitaceae*, native to Africa, parts of Middle East as well as drier parts of India [1] Found in many kinds of habitat and different types of soils in Sudan, it is well-distributed from northern Saharan region across to the southern Savannah.

Different parts of *B. aegyptiaca* have been reported to posses miscellaneous medicinal uses such as treatment of syphilis, treating abscess, leucoderma, malaria, wounds, colds, aches, liver and spleen disorders, also reported to be an antihelminthics, as purgative, vermifuge, and febrifuge [2]. The bark of the plant is also useful in treating mental illness, epilepsy, yellow fever, jaundice and syphilis. It can act as a fumigant to heal circumcision wounds [3]. In addition its use to treat round worm infections, as well as for fish venom is well documented [4]. Balanitin-6 and balanitin-7 extracted from *Balanite aegyptiaca* kernels exhibited significant anticancer in vitro against human cancer cell lines (A549 non-small cell lung cancer and U373 glioblastoma), also both compounds showed profound anticancer in vivo and increased the survival rate of mice bearing murine L1210 leukemia with efficacy comparable to vincristine [5] Saponins isolated from *B. aegyptiaca* demonstrated potent anticancer effect against human tumor cell lines (breast cancer cells (MCF-7) and colon cancer cells (HT-29), the isolated saponins manifested their anticancer activity in halting cell proliferation with high selectivity towards cancer cells compared to normal cell lines [6]. In recent study balanitoside extracted from the fruit of *B. aegyptiaca* and evaluated for its antitumor efficacy in vivo on Swiss albino mice bearing Ehrlich ascites carcinoma (EAC), the active saponin excelled itself to reduce the number of EAC cells and prolong life span of treated animals [7]. The stem bark and roots of *B. aegyptiaca* was reported to be rich in saponins, steroidal saponins and yamogenin [8]. Thus, it’s tempting to speculate that stem bark extract(s) conform same or even better antitumor efficacy.

Chemotherapeutic agents are typically natural products or their synthetic analogs that block or delay the progression of cancerous cells by modulation of cell proliferation and differentiation. The vast majority of drugs currently used in chemotherapy are designed to selectively interfere with rapidly proliferating cells [9].

Angiogenesis is defined as the formation of new blood vessels from the pre-existing vasculature. In healthy adults, angiogenesis induction and termination is tightly regulated by pro- and antiangiogenic factors which facilitates controlled physiological processes like wound healing, female reproductive cycle and the general maintenance of tissue homeostasis [10]. However pathological angiogenesis may also occur in cancer, inflammatory diseases, and degenerative conditions such as glaucoma, age related macular degeneration among others [11]. The induction of angiogenesis in the tumor context is due to an imbalanced ratio between tumor growth rate and metabolic demand which leads to insufficient supply of oxygen and nutrients. To cope with resulting hypoxia, tumors activate transcription factors like hypoxia inducible factor (HIF) [12]; pro-angiogenic growth factor such as vascular endothelial growth factor (VEGF) and other growth factors (e.g. Matrix Metalloprotease 9, MMP-9). These factors as a result stimulate the neovascularization process which consequently leads to tumor growth and metastasis. Therefore inhibition of tumor angiogenesis has become a fundamental approach in cancer therapeutics [13]. Most of the approved antiangiogenic drugs are designed to interfere with one or more of pro-angiogenic signaling cascades, most commonly with the VEGF pathway [14]. VEGF is a signal protein that stimulates various steps in angiogenesis cascade such as cell proliferation, migration and cell survival of endothelial cells [15]. When solid tumors grow in size, the demand for oxygen increases causing release of oxygen free radical which in turn stimulate vascular endothelial growth factor (VEGF) that trigger tumor angiogenesis, it’s reported that VEGF level is upregulated in RNA and protein of many types of malignancy [16].

From the earliest times *B. aegyptiaca* has been prized for its curative properties to several diseases as a home remedy. Many pharmacological studies have been carried out to support most of these traditional claims. To the best of our knowledge this is the first study to evaluate its stem bark extracts for antiangiogenic, antitumor and cytotoxicity properties.

## Methods

### Plant material

Plant material was collected during the period of March-July 2014 from Shandi, River Nile state, Sudan. The taxonomic identification of this plant was carried out at the Medicinal and Aromatic Plants Research Institute (MAPRI), National Center for Research-Sudan by Dr. W.E.A/Alla, and the voucher specimen (Ref. No. 30593/2014) was deposited at the herbarium of the institute.

### Preparation of extracts

The dried stem bark was ground to powder; the plant material was extracted successively with n-hexane, chloroform, methanol and water using maceration method as described previously [17, 18]. All the extracts were prepared by 250 ml of the solvents using maceration method (40 °C) with shaking. The extracts were filtered; evaporated and stock solutions of the extracts were prepared at 10 mg/ml in dimethyl sulfoxide (DMSO). The stock solutions as well as DMSO (vehicle) were diluted with cell culture medium, so the highest DMSO concentration exposed to the cells or implanted tissue was 1 % *v/v*.

### Determination of plant extract yield

The yields of the stem bark extracts of *B. aegyptiaca* based on dry weight basis were calculated from the following equation:

$$ \mathrm{Yield}\kern0.5em \left(\mathrm{g}/20\;\mathrm{g}\kern0.5em \mathrm{of}\kern0.5em \mathrm{dry}\kern0.5em \mathrm{plant}\kern0.5em \mathrm{material}\right)=\mathrm{W}1/\mathrm{W}2\times 100 $$

Where, W1 and W2 were the weight of the extract after the solvent evaporation and the weight of the dry plant material, respectively.

### Chemicals and reagents

Endothelial Cell Medium (ECM) supplied with endothelial cell growth supplements (ECGS) was purchased from ScienCell, USA. Cell culture RPMI 1640 medium and Dulbecco’s Modified Eagle Medium (DMEM) were purchased from GIBCO; Trypsine and heat inactivated foetal bovine serum (HIFBS) were obtained from GIBCO, UK. MTT reagents, suramin, aprotinin, 6-aminocarpoic acid, L-glutamine, thrombin, gentamicin were purchased from Sigma-Aldrich, USA.

### Cell lines and culture conditions

Human umbilical vein endothelial cells (HUVECs), human colorectal carcinoma cell line HCT-116, human hormone sensitive and invasive breast cancer cell line MCF-7, human leukemia K562 and U937 were purchased from ScienCell, USA. HUVECs were maintained in ECM medium supplemented with 5 % HIFBS, 1 % PS and 1 % ECGS, whereas HCT-116, K562 and U937 were maintained in RPMI; MCF-7 cell line was maintained in DMEM. Cells were cultured in a humidified incubator at 37 °C supplied by 5 % CO_2_.

### Experimental animals

The 12–14 weeks old Sprague Dawley male rats were obtained from animal house facility of USM. The rats were kept in well ventilated cages at 12 h light with food and water in animal transient house (School of Pharmaceutical Sciences, USM) for one week prior to the experiment. Athymic NCR nu/nu nude mice were obtained from Taconic Farms Inc., USA. The mice of the same gender were housed in specific pathogen-free (SPF) cages supplied with high efficiency particulate air (HEPA) filters. Free access to autoclaved food and water was provided and the autoclaved bedding was changed twice weekly. The procedures were approved by the USM Animal Ethics Committee with a reference number USM/Animal Ethic approval/2012/ (81) (475).

### *Ex vivo* rat aortic ring assay

The Rat aortic ring assay was used according to techniques established by [19] with slight modification. In brief, aortic rings (1 mm thickness) taken from thoracic aortas of 10–12 weeks old male Sprague Dawley rats were seeded individually in 48-wells plate in 300 μL serum free M199 media containing 3 mg/mL^−1^fibrinogen and 5 mg ml^−1^aprotinin. 50 NIHUmL^−1^thrombin in 0.15 M NaCl were added in each well. After 90 min incubation at 37 °C, various concentrations of each extract were dissolved in 0.3 ml M 199 medium supplemented with 20 % HIFBS, 0.1 % έ-aminocaproic acid, 1 % L-Glutamine, 2.5 μg/mL amphotericin B, and 60 μg/mL gentamicin were added to each well. Suramin used as positive and DSMO as a negative controls. On the fourth day, the medium was replaced with a fresh one containing the extracts. On fifth day, aortic rings were photographed at 4x magnification using an inverted light microscope (EVOS). The angiogenic response was determined by measuring the distance of blood vessels outgrowth from the primary tissue ex-plants using the same instrument with the aid of Leica Quin software package [20]. Results are presented as mean percent inhibition to the negative control ± SD, (*n* = 3).

### Cell proliferation assay

Cytotoxicity of extracts was evaluated by MTT assay. Cells were treated for 48 h with different extracts of *B. aegyptiaca* or 1 % DMSO as a negative control. Viability of cells was determined by MTT test as described previously [21]. Assay plates were read using a microplate reader (Tecan infinite bro 2000) at absorbance 570. The results are presented as percentage inhibition to the negative control (*n* = 3).

### Migration assay

The assay was carried out as described previously [22]. In brief, HUVECs were seeded in 6 well plates till the formation of a confluent monolayer after which a wound was created using 200 μl micropipette tip. The detached cells were removed by washing with PBS and the plates were treated with (MBA). The wounds were photo-graphed after 12 and 18 h, and the width of the cell-free wounds was measured using an inverted light microscope supplied with Leica Quin computerized imaging system. Ten fields per well were photographed and minimum of 30 readings per field were taken. The results are presented as mean percentage of migration inhibition compared to control ± SD, (*n* = 3).

### Determination of VEGF levels

Concentration of human VEGF in HUVEC cells lysates was determined by human VEGF ELISA kit (China) according to manufacturer’s instructions. The kit consists of anti-human VEGF-1 mouse IgG monoclonal antibody and a horseradish peroxidase conjugated secondary antibody and recombinant human VEGF 165 as a standard. HUVEC cells were seeded in 6-well plates at 1 × 10^6^ in 3 mL of ECM medium. After overnight attachment, the cells were treated for 6 h with (MBA) at 50 and 100 μg/ml. Calibration curve of VEGF standard was prepared simultaneously with the samples, and concentration of VEGF-1 in cell lysates was determined by applying the linear regression equation, (y = 0.004x + 0.3114, R^2^ = 0.982).

### Tube formation assay

The ability of HUVECs to form tube-like structures was assessed on a matrigel matrix. Briefly, the matrigel matrix was allowed to polymerize for 45 min at 37 °C and 5 % CO_2_. HUVECs were trypsinized and seeded (3 × 10^4^ cells per well) in 100 μl of ECM containing various concentrations of (MBA) in triplicates. After 6 h tubular structures were imaged under an inverted light microscope at 4X magnification. The quantitative assessment of tube formation inhibition was achieved by measuring the area occupied by tubular structures using the Scion Image analysis program [23]. The results are presented as a mean percentage of inhibition ± SD, (*n* = 3).

### Determination of total phenolic content

Total phenolic contents in the four extracts of stem bark were determined using Folin-Ciocalteu reagent according to previously described method [24]. Twenty μl of extract and gallic acid solutions were pipetted into separate test tubes followed. Then, 1.58 ml of distilled water and 100 μL of 2 N Folin-Ciocalteu reagent were added and mixed thoroughly. After that, 300 μl of 20 % sodium carbonate solution were added and the mixture was incubated for 2 h at room temperature (22–25.6 °C). The absorbance was measured at 765 nm using a Hitachi U-2000 spectrophotometer (Hitachi, Japan). The phenolic content was expressed as mg of gallic acid equivalent/g extract.

### Determination of total flavonoids

The total flavonoids contents in the four extracts of stem bark were determined using aluminum chloride colorimetric method with quercetin as a standard [25]. A solution of 6 mg/mL of *B. aegyptiaca* extracts in 80 % methanol and different concentrations of quercetin (0.007, 0.015, 0.0313, 0.0625, 0.125, 0.25, 0.5, and 1 mg/ml in 80 % methanol) were prepared. 500 μl of plant extracts and each concentration of quercetin (Sigma Aldrich, Germany) were pipetted into respective test tubes followed by 0.1 ml of 10 % (*w/v*) aluminum chloride (R & M Chemicals, UK), 0.1 ml of 1 M potassium acetate (Merck, Germany), 1.5 ml of methanol and 2.8 ml of distilled water. The test tubes were thoroughly mixed and after incubating at room temperature (24 to 26 °C) for 30 min, the absorbance of the reaction mixture was measured at 415 nm with a Tecan infinite pro-2000 spectrophotometer against blank. The amount of 10 % (*w/v*) aluminum chloride was substituted by the same amount of distilled water in a blank. The concentration of total flavonoids contents of the extracts were determined using a standard curve and quercetin was used as a standard. The data were presented as mean ± SD (*n* = 3).

### DPPH free radical scavenging activity

The DPPH radical scavenging activity of the samples was calculated as described by Molyneux 2004 [26] with some modification, in brief, 100 μl of each extract or scorbic acid (prepared in methanol) were added to 100 μl of DPPH radical dissolved in methanol (200 μM), and the reaction mixture was incubated for 30 min. Then, the absorbance of the mixture was measured at 517 nm, which shows the amount of DPPH radical remaining in the solution. The scavenging activity expressed as the IC_50_ (represents the sample concentration required to scavenge 50 % of DPPH free radicals) and calculated by using the following formula:

$$ \%\kern0.5em \mathrm{inhibition}=\left[\left(\mathrm{A}\mathrm{c}-\mathrm{A}\mathrm{s}\right)/\mathrm{A}\mathrm{c}\right]\times 100 $$

Where Ac is the absorbance of the control, and As is the absorbance of sample.

### Ferric reducing antioxidant power (FRAP) assay

The reducing power of the extracts was measured using a modification of the FRAP assay conducted by Benzie and Strain in 1999 [27]. The FRAP reagent was prepared by mixing 2.5 mL of a 10 mmol/L TPTZ solution in 40 mmol/L HCl with 2.5 ml of 20 mmol/L FeCl_3_ · 6H_2_O and 25 mL of 0.3 mmol/L acetate buffer (PH 3.6). 20 μl of the plant extract or standard was mixed with 150 μL of FRAP reagent prepared freshly and the reaction mixture was incubated at 37 °C for 4 min. Absorbance at 593 nm was read against the blank (methanol). Calibration curve was determined by using different concentrations in the range of 0.003–0.12 mg/ml (FeSO_4_ · 7H_2_O) and by applying a standard curve equation (Y = 4.2669x + 0.1814) (R^2^ = 0.992).

The data were represented as ferrous sulfate [Iron (II) sulfate] equivalent μmol/ml. Sample determinations were all performed in triplicate.

### ABTS radical scavenging assay

ABTS radical scavenging activity of extracts was determined as previously described [28]. Initially, ABTS stock solution containing 5 ml of 2.45 mM potassium persulfate was mixed with 5 ml of 7 mM ABTS+ solution to produce ABTS radical cation (ABTS+). Then, the mixture was allowed to stand in the dark at room temperature for 12–16 h prior to use. Methanol was used to dilute the ABTS+ solution to a final concentration that would give an absorbance of 0.70 ± 0.02 at 734 nm. About 100 μl of extracts was added to 900 μl of ABTS solution. This solution was vortexed for 15 s and the absorbance was measured at 734 nm after 6 min using a UV visible spectrophotometer (Hitachi U-2000; Hitachi, Tokyo, Japan). Vitamin C was used as reference standard at final concentrations in range of 0.025 to 1 mg/ ml. The scavenging activity of test sample was calculated and expressed as the IC_50_ using the following formula:

$$ \%\kern0.5em \mathrm{inhibition}=\left[\left(\mathrm{A}\mathrm{c}-\mathrm{A}\mathrm{s}\right)/\mathrm{A}\mathrm{c}\right]\times 100 $$

Where Ac is the absorbance of the control, and As is the absorbance of sample.

### In vivo antitumor study

HCT-116 human colorectal carcinoma cells was selected as a model of colon cancer [29]. The cells were propagated in RPMI 1640 medium with 10 % FBS and 1 % PS solution. Eighty percent confluent HCT-116 cells cultures in T75 flasks were trypsinized and re-suspended in 10 ml fresh medium, the cells were collected by centrifugation at 1000 rpm for 5 min and washed with a sterile PBS. The nude mice with aged 5–7 weeks were injected subcutaneously in the right flank with 5 × 10^6^ cells in 200 μl culture medium using 1 ml insulin syringe (27 G needle).

After one week of tumor initiation, animals were divided randomly into four groups of 6 animals each. Group 1 received 0.2 ml distilled water (control), and Groups 2, 3 and 4 received oral treatments with 400, 200 and 100 mg/kg bodyweight of (MBA), respectively. The tumor size and body weight were recorded before starting the treatment and at weekly basis. Treatment of animals was performed orally by oral gavages (wt/wt) once a day for a period of 5 weeks. The tumor dimensions were measured by a caliber in 2 angles, length and width as well as depth [30]. The tumor size was calculated by applying the formula: Tumor volume (mm3) = (W + L) / 2) ^ 3) × 2 Where W is the width and L is the length. Also following value was calculated :% ∆T/∆C, where, ∆T = T − ∆_0_ and ∆C = C − ∆_0_ (∆_0_ is the average tumor volume at the beginning of the treatment, T and C are the tumor volumes at a specified day for treated and control groups, respectively). Generally, the ∆T/∆C value in percent is used as an indication of antitumor effectiveness, and a value of ∆T/∆C ≤42 % is considered as significant antitumor activity by the Division of Cancer Treatment, NCI, NIH [31].

### Statistical analysis

Results were presented as means ± SD and differences between groups were compared by the one way ANOVA and considered significant at *P* < 0.05, 0.01 or 0.001. The statistical analysis was carried out by using SSPS edition 20.

## Results

### Plant extraction

Four exacts were prepared from stem bark of *B. aegyptiaca*, starting with n-hexane followed by chloroform, Methanol and water. The texture and the weight of each extract were reported and the results are presented as a wt/wt percent yield.

Hexane extract of *B. aegyptiaca* stem bark showed the lowest yield (0.107 %), while methanol extract produced highest amount (0.959 %) followed by water extract (0.646 %) and chloroform extract (0.216 %).

### Rat aorta ring assay (antiangiogenic)

**Fig. 1:**
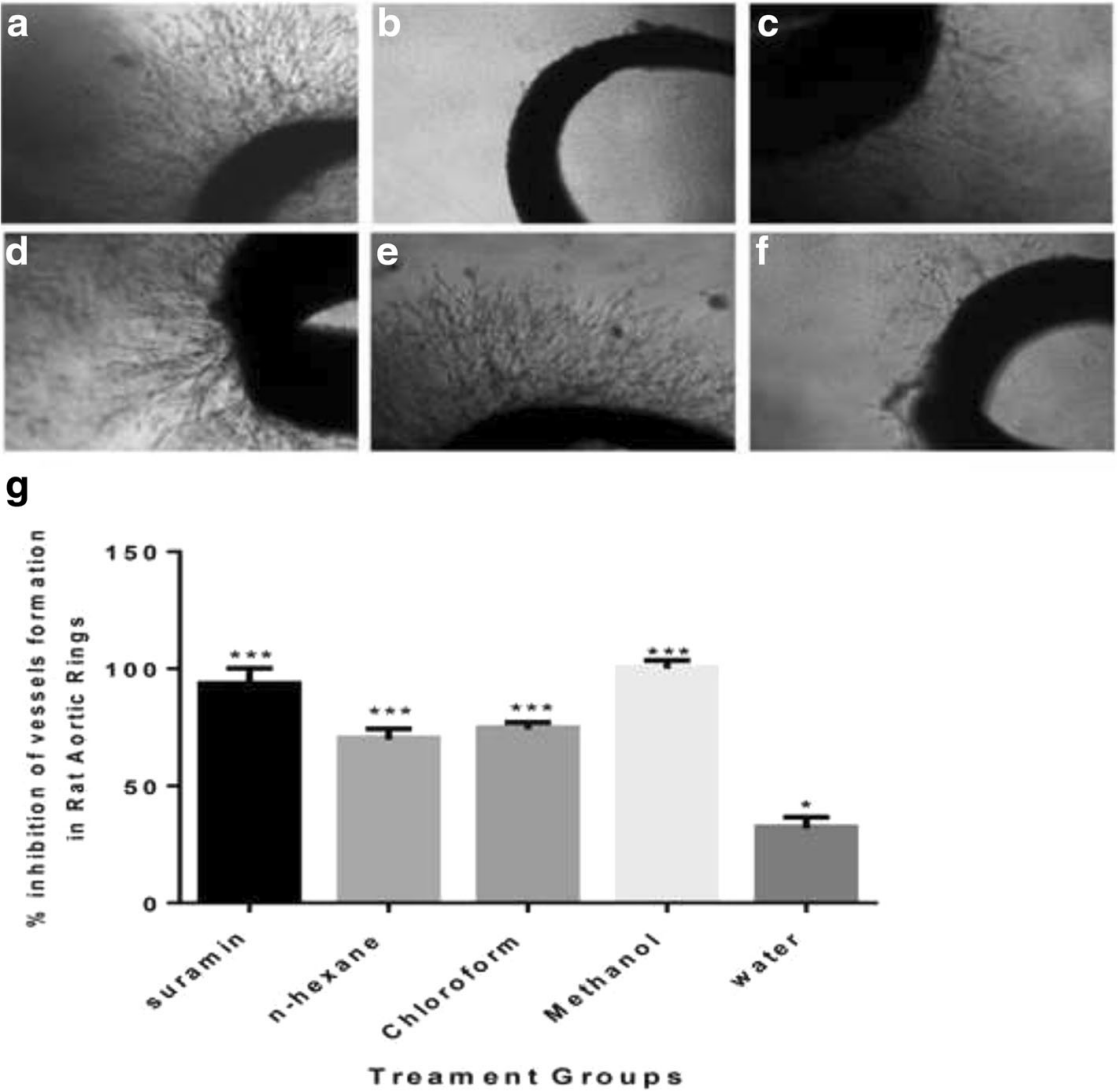
Effect of different extracts on micro vessels formation in *ex vivo* rat aorta ring assay, **a** explants treated with 1 % DMSO (vehicle), **b** tissue treated with methanol, **c** chloroform, **d** n-hexane and **e** water extract of *B. aegyptiaca* stem bark, while **f** is Suramin as a positive control. (Images were taken under an inverted phase-contrast microscope at 4X). **g** Graphical representation of the inhibitory effect tested extracts on blood vessel growth in rat aortic rings at concentrations of 100 μg/mL. The result is compared with the standard reference, suramin. All values are represented as the mean ± SD (*n* = 10), and *** indicates significant differences of *p* < 0.001 compared with the control-treated group (DMSO)

This assay was performed as the primary assay to study the antiangiogenic properties of the plant extracts in rat aorta model. Figure 1 illustrates the microvessels outgrowth in untreated aortic ring as well as treated with different extracts of *B. aegyptiaca*. Table 1 shows antiangiogenic properties of four extracts by rat aorta ring assay. The result shows that all organic solvent extracts decreased the growth of micro-blood vessels, nevertheless methanol extract stand as potent antiangiogenic with 100 % inhibition of blood vessel (at 100 μg/ml), followed by chloroform extract with inhibition 74.05 ± 0.07 %, while the positive control, suramin as antiangiogenic agent inhibited the growth of blood vessel by 92.88 ± 0.06 %. Thus, methanol extracts was subjected to further in vitro and in vivo studies.

**Table 1:** The rat aorta ring assay result, the results are presented as a mean percent inhibition to the vehicle (DMSO) ± (*n* = 3)

Extract	Inhibition %
Hexane	69.66 ± 0.18
Chloroform	74.05 ± 0.07
Methanol	100 ± 0.00
Water	31.87 ± 0.08
Suramin	92.88 ± 0.06

### Cytotoxicity

**Fig. 2:**
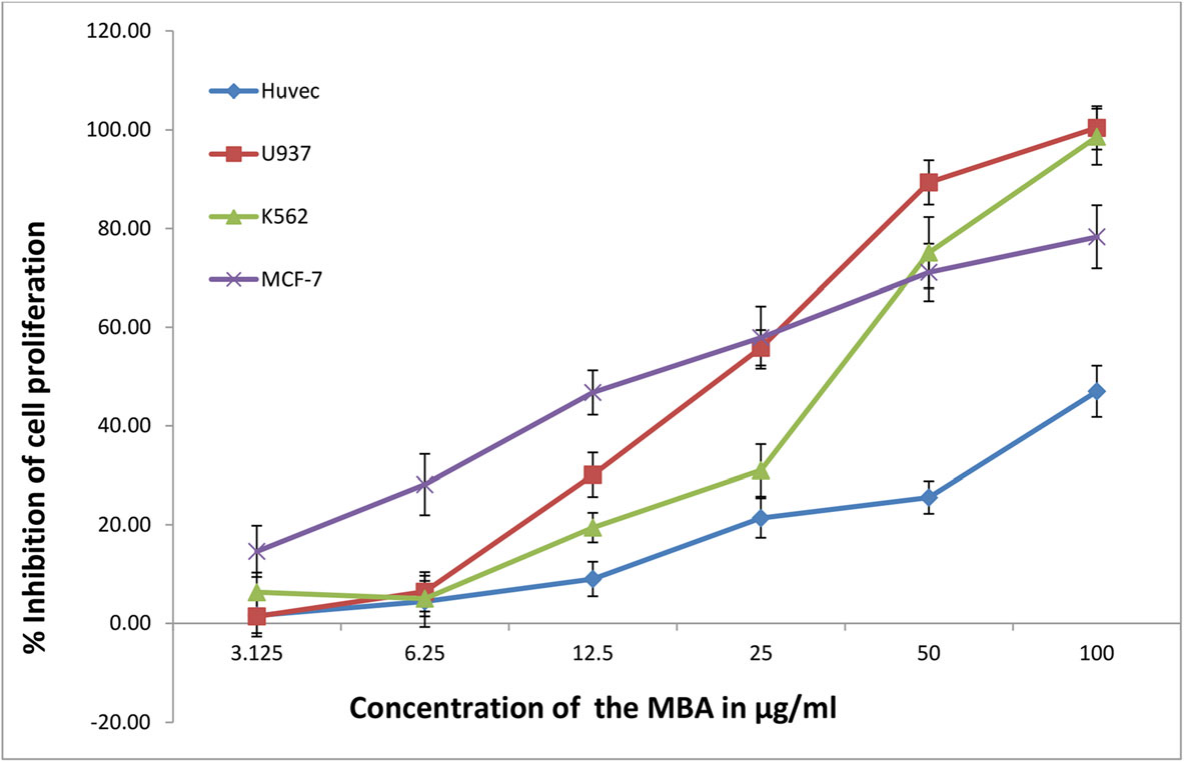
Dose-dependent antiproliferative effect of methanol extract of *B. aegyptiaca* stem bark on U937, K562, MCF-7 and HUVEC cell lines was assessed using MTT assay. Values are expressed as mean ± SD (*n* = 6)

The cytotoxic effect of *B. aegyptiaca* was evaluated on HUVECs versus four cancer cell lines namely HCT-116, K562, U937 and MCF-7. The median inhibition concentration (IC_50)_ from dose dependent curve equations was calculated for all tested extracts (Table 2). The methanolic extract was safe on HUVECs with IC_50_ = 72.86 μg/ml. Interestingly, it demonstrated selective cytotoxicity against leukemia cell lines K562 and U937 with IC_50_ 26.11 and 15.55 μg/ml respectively as well as against breast cancer cell line MCF-7 with IC_50_ 19.75 μg/ml (Fig. 2). It exhibited moderate antiproliferative effect against colon cancer (HCT-116) with IC_50_ = 38.28 μg/m. Water extract also was safe on HUVECs and showed antiproliferative effect against colon cancer cell (HCT-116) and leukemia (K562) with IC_50_ = 46.23 and 26.3 μg/ml respectively, while hexane and chloroform extracts showed moderate cytotoxicity against tested cancer cell lines.

**Table 2:** Cytotoxicity effect of *Balanite aegyptiaca* stem bark extracts on tested cell lines

Solvent used	Cells inhibition (IC_50_ μg/mL)				
	HCT116	K562	U937	MCF-7	HUVCs
Hexane	65.27	77.24	54.63	55.3	52.11
Chloroform	52.75	72.3	24.12	48.37	30.1
Methanol	38.23	26.11	15.55	19.75	72.867
Water	46.23	26.3	23.03	64.93	46.2

### Cell migration assay

**Fig. 3:**
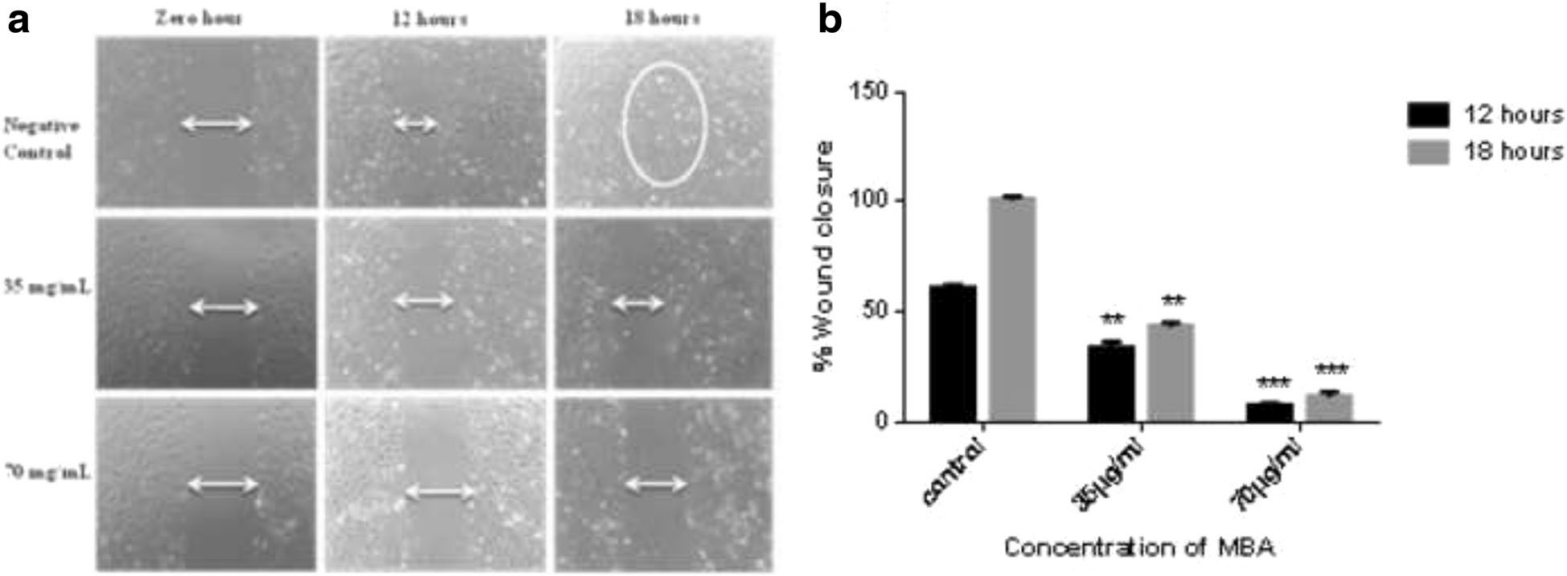
Effect of methanol extract on human umbilical vein endothelial cells (HUVECs) migration. **a** A wound was created and the cell treated with 1 % DMSO (negative control) or 35 μg/mL and 70 μg/mL of methanol extract of *B. aegyptiaca*. Pictures of the wound were captured at zero, 12 and 18 h, under magnification 4X. **b** Graphical representation of the inhibitory effect of methanol extract on HUVECs migration (wound closure)

Endothelial cell migration towards the stimulant is a vital step in formation of new blood vessels. The effect of (MBA) on endothelial cell migration was assessed using endothelial cell migration wound healing assay. Significant reduction in HUVECs motility was achieved at non-toxic doses, at concentration 35 μg/ml the extract inhibited migration of the cells by 61.8 ± 1.8 % and 48.7 ± 2.31 % after 12 and 18 h treatment, respectively (*P* < 0.05) (Fig. 3). At 70 μg/ml it inhibited HUVECs migration by 88.1 ± 3.1 % and 83.5 ± 1.28 % after 12 h and 18 h incubation period, respectively (*P* < 0.001) Fig. 3.

### Effect *B. aegyptiaca* methanol extract on VEGF expression

**Fig. 4:**
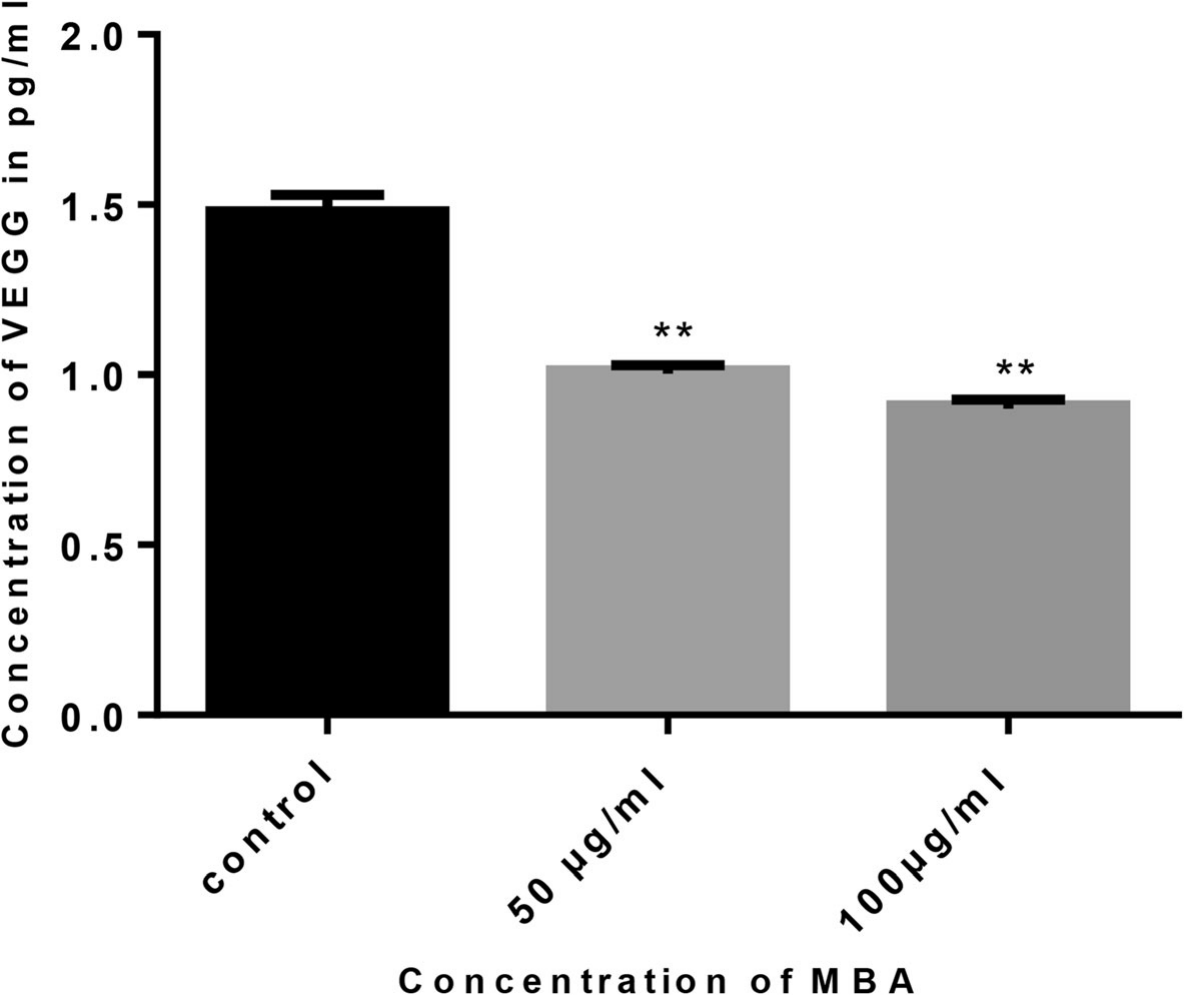
Graphical representation of VEGF level reduction in HUVECs lysates by methanol extract of *B. aegyptiaca* stem bark

To assess the mechanism of action for antiangiogenic property *B. aegyptiaca*, we studied the effect of (MBA) on expression of VEGF165 on HUVECs. It significantly reduced the level of VEGF compared to untreated cells (*P* < 0.01). At concentration 50 and 100 μg/ml methanol extract inhibited the VEGF by 31.807 and 38.76 % respectively, the VEGF concentration in HUVEC cell lysate treated with (MBA) at 50 μg/ml was 1.009 pg/ml and at 100 μg/ml was 0.9 pg, while it was 1.47 pg/ml in untreated cells (Fig. 4).

### Tube formation assay

**Fig. 5:**
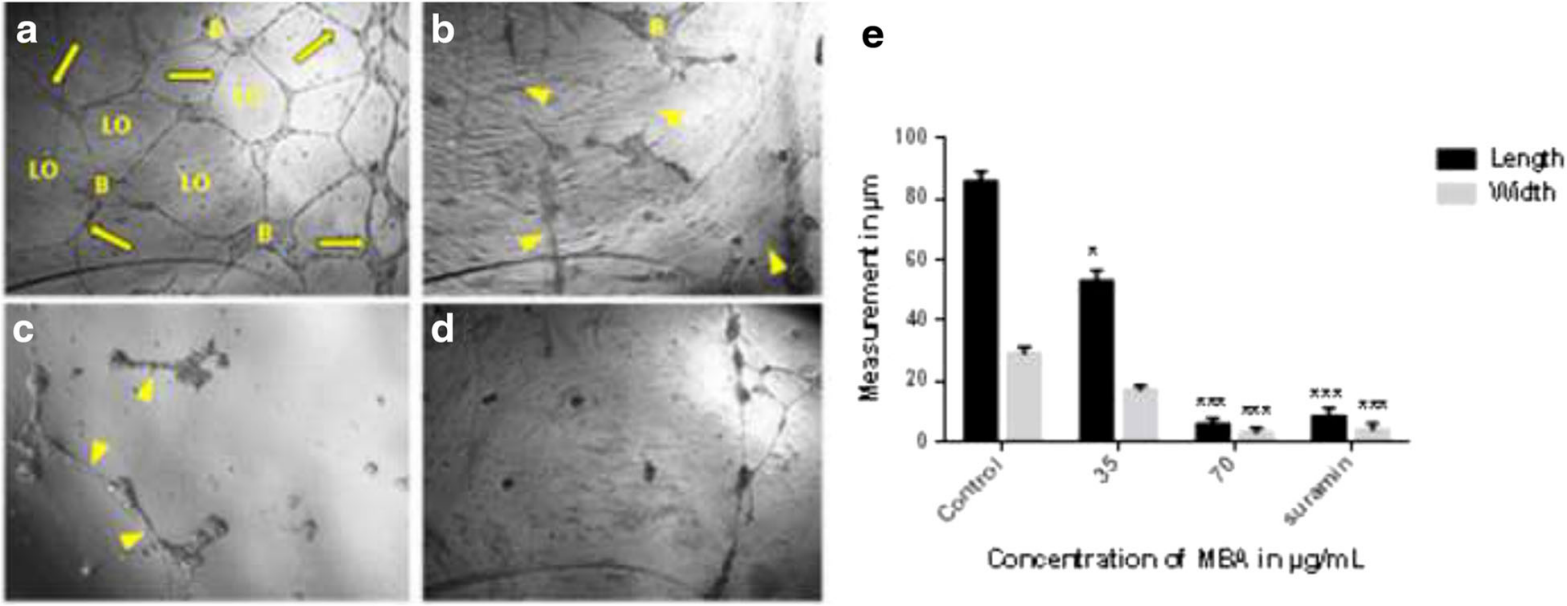
Effect of methanol extract of *B. aegyptiaca* on tube formation of human umbilical vein endothelial cells HUVECs on Matrigel. Phase-contrast micrographs showing the effects of MBA and suramin on the differentiation of HUVECs are presented. **a** Control group (vehicle treated 1 % DMSO), depicting that the endothelial cells grown on the three-dimensional Matrigel media differentiated into branching structures to form capillary tube-like structures composed of multiple cells with intercellular spaces or lumens. The image clearly exhibits prominent areas covered tubes (arrows), loops (Lo), and branching points (b). **b** Treatment with MBA (35 μg/mL) caused disruption of bridges (arrow head) and branching points. **c** Treatment with MBA (70 μg/mL) caused notable disassembly in the tube-like structures. **d** Treatment with suramin (100 μg/ml) caused more than 90 % abrogation of the bridges in the tube-like structures and branching points. Images were taken under an inverted phase-contrast microscope at4X). **e** Graphical illustration of the dose-dependent inhibitory effect of MBA on the length and width of the capillary-like structures in HUVECs. All values are represented as the mean ± SD (*n* = 6); * and *** indicate significant differences of *p* < 0.05 and *p* < 0.001, respectively, compared with the control-treated group

Endothelial cells in normal setting inclined to form tube-like structure network in matrigel within 6 h. Treating HUVECs with different concentrations of (MBA) inhibited tube formation in a dose-dependent manner. At lower concentration methanol extract reduced the area covered by network structures and broken tubules were formed. At 35 μg/ml (MBA) degraded the tube-like structures, reducing the length and width of network; at 70 μg/ml it completely abrogated endothelial tube formation (Fig. 5B-C). Figure 5E depicts the dose-dependent inhibitory effect of MBA on the width and the length of the human endothelial cell tube network (μm).

### Antioxidant evaluation

Antioxidant effect of *B. aegyptiaca* stems bark on DPPH and ATBS scavenging activities, in addition to soluble total phenolic and flavonoids contents are shown in Table 3. Result shows that methanol extract displayed highest level of phenolic contents 35.17 mg GAE/g, followed by chloroform 19 mg GAE/g, water 10.54 mg GAE/g and hexane 8.9 mg GAE/g. Chloroform extract showed considerable amount of flavonoids contents 141.12 mg quercetin equivalent/g, followed by methanol extract 112.83 mg quercetin equivalent/g, hexane and water extracts exhibited moderate content of flavonoids (57.11 and 28.7 mg quercetin equivalent/g respectively). Methanol extract scored the highest free radical activity with IC_50_ = 40 μg/ml, in DPPH assay, chloroform extract showed relatively low antioxidant with IC_50_ = 181.17 μg/ml, while hexane and water extracts showed neglectable antioxidant activity (Table 3). In addition, methanol extract exhibited the highest values in FRAP 0.52 (FeSo4 μmol/ml), moreover it showed highest antioxidant activity on ABTS assay with IC_50_ = 125.85 μg/ml assays.

**Table 3:** Total phenolic and flavonoid contents and antioxidant activities of different plant extracts

Extract	FRAP (FeSo4) μmol/mL	ABTS (IC_50_) μg/mL	DPPH (IC_50_) μg/mL	TFC (mg/g)	TPC (mg/g)
Hexane	0.27	205.56	>500	52.11	8.97
Chloroform	0.31	143.53	281.17	141.12	19.84
Methanol	0.52	125.85	40.08	112.83	35.18
Water	0.24	165.75	>500	28.71	10.55
Ascorbic acid	2.25	3.75	3.12	------	------

Strong positive correlations were detected between TPC and TFC against ferric reducing antioxidant power; in addition good negative correlations were detected between TPC and TFC against DPPH and ABTS (Table 4).

**Table 4:** Relationship between TPC and TFC with antioxidant assays

Antioxidant assay	FRAP	DPPH	ABTS
TEST			
TPC	0.949**	−0.417*	−0.521*
TFC	0.687**	−0.428*	−0.459*

*&** means correlation is significant at the 0.05 and 0.01 levels (2-tailed), respectively

### Antitumor activity of *B. aegyptiaca* stems bark

**Fig. 6:**
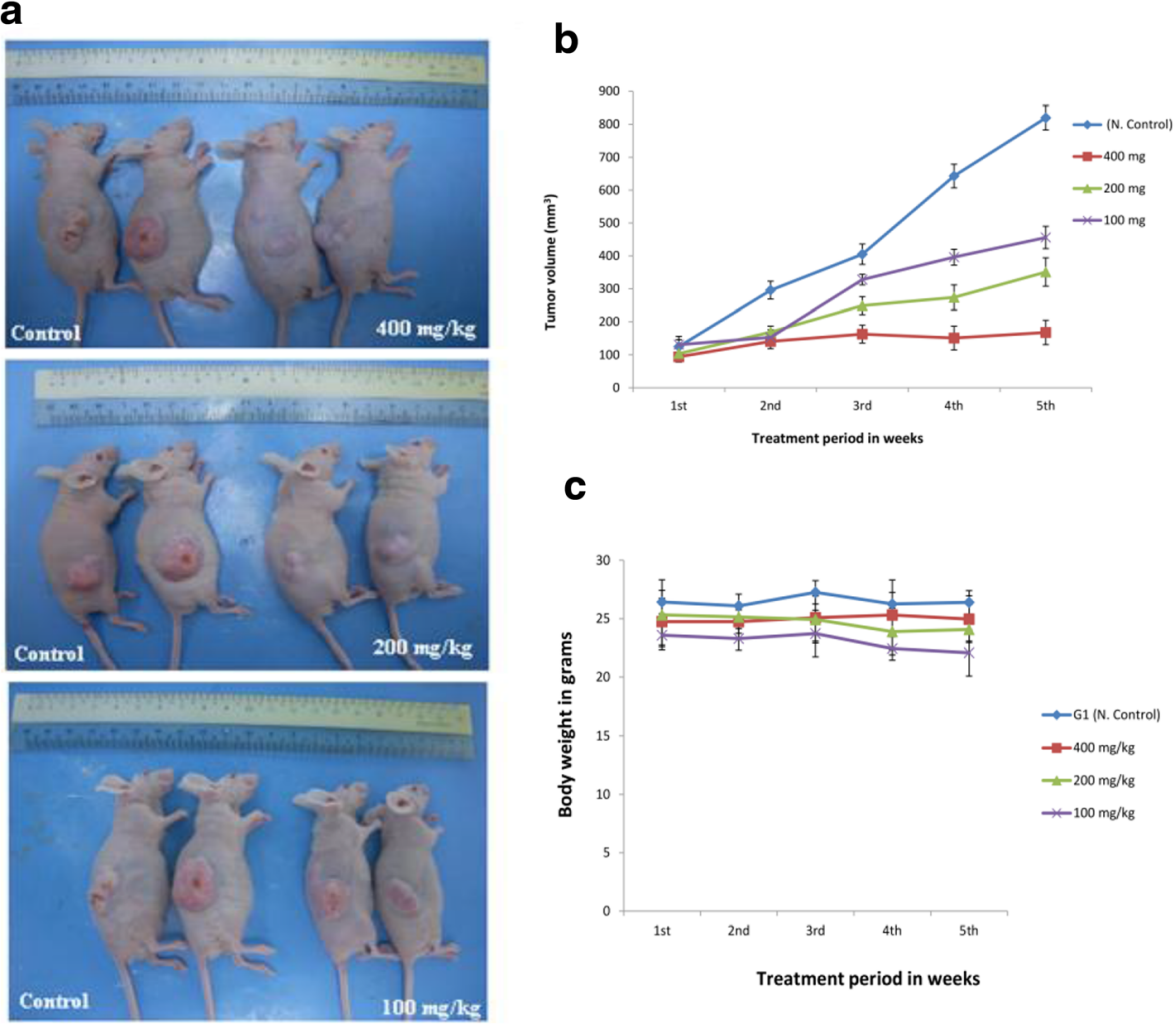
In vivo antitumor activity in nude mice induced with HCT-116 tumor at four five weeks post-inoculation day. **a** Animals untreated as well as treated with 400, 200 and 100 mg/kg, and untreated group (Control). **b** Effect of MBA on the average tumor growth. **c** Effect o MBA on body weight of treated animals. All values are expressed as mean ± SD (*n* = 6).^∗^ *P* < 0.05, ^∗∗^ *P* < 0.01

Xenograft models of human neoplasic play an important role in the screening and evaluation of candidates for new antitumor agents. (MBA) inhibited the growth of new blood vessels from explants rat aorta ring assay in *ex-vivo* model, besides it showed selective antiproliferative effect against colon cancer (HCT-116), therefore it worthily to be evaluated for antitumor in vivo*.* The NCR NuNu nude mice were injected with HCT-116 cells according to the method developed by Cheon and Orsulic (2011) [32]. The deficiency in T cell function allows athymic mice to accept and grow xenografts as well as allografts of normal and malignant tissues. Animals bearing tumor treated with (MBA) showed dose dependent inhibition of tumor growth, Fig. 6 illustrates the tumor growth in respective tested groups. A significant ∆T/∆C = 8.57 % antitumor activity of (MBA) (at a dose of 200 mg/kg) on 5 week post-cell inoculation day was observed. At a dose of 400 mg/kg it showed profound activity (∆T/∆C = 1.84 %, *P* < 0.001). There was no toxicity effect of (MBA) on body weight of treated animals (Fig. 6c).

### Histological assessment

**Fig. 7:**
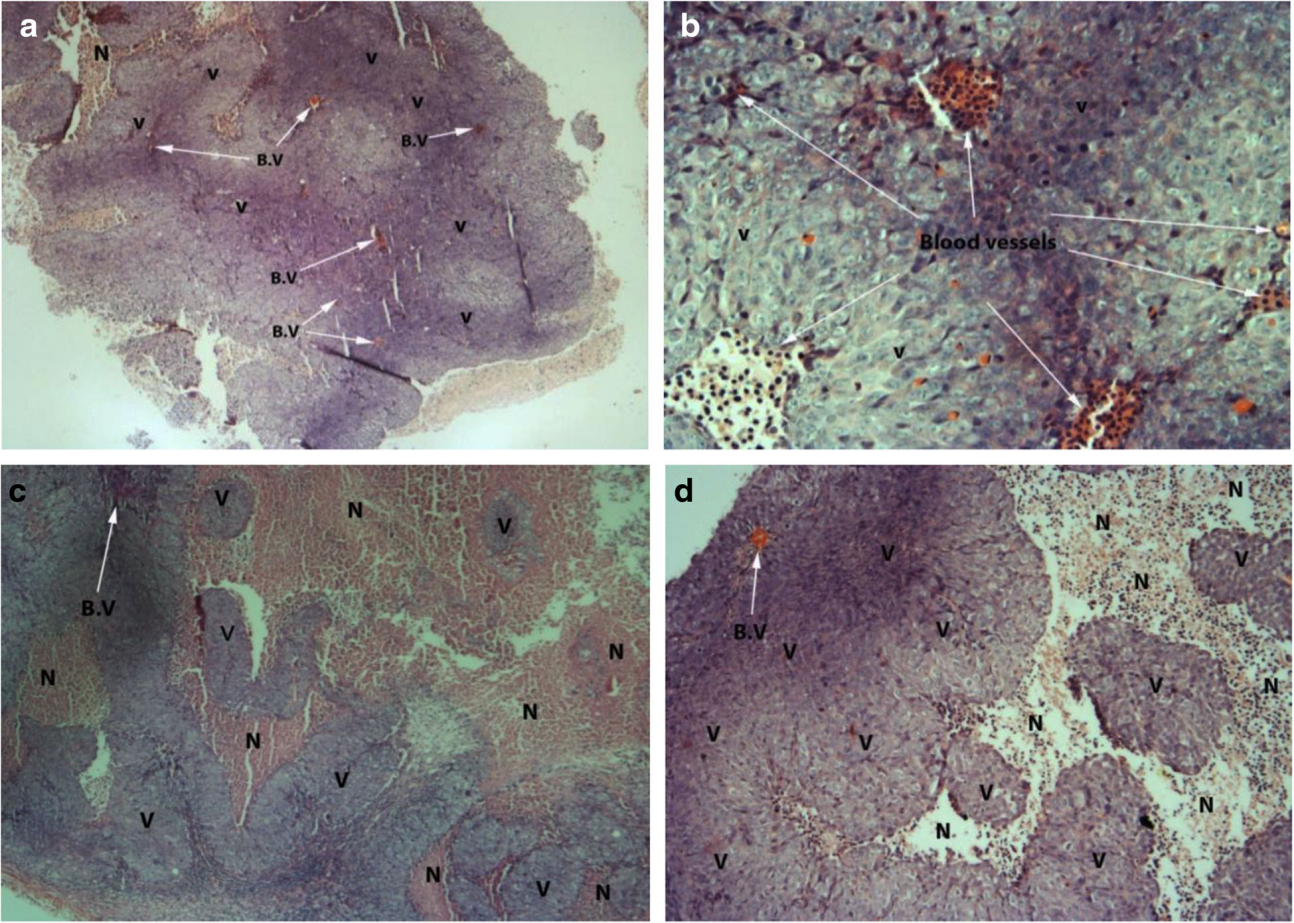
Cross section at tumor tissue. The tumor cross sections were stained with hematoxylin/eosin, and the number of the intratumor blood vessels was counted as well as extent of apoptosis/necrosis. **a**, **b** Tumor tissue excised from untreated animals, the pictures were captured at 4 × and 20 × magnifications, respectively. The extents of the apoptotic/necrotic areas were less than in the treated groups. **c** Tissue excised from animals treated with MBA 200 mg/kg (at 10x). **d** Tumor specimen from animals treated with 400 mg/kg (at 10 x). (V) refers to viable tumor cells, and (N) refers to necrotic/apoptotic tumor cells

Histological study exhibited notable antiangiogenic effect of MBA *via* reducing tumor vasculature. Figure 7a) and b) show the tumor tissue excised from untreated animals in which there is no or minor necrosis and the tumor cells showing compactness with adequate supply of blood vessels. The specimens of the tumors excised from animals treated with different doses of MBA showed clear loss of cell compactness with areas of decreased cell density in the tumor due to severe necrosis.

## Discussion

Medicinal herbs have been used traditionally for hundreds of years to treat various types of human illness. The impact on cancer treatment has become increasingly crucial and important as more patients tend to resort to herbal alternatives than conventional chemotherapy. A large number of botanical herbal extracts and their major constituents have been evaluated for cancer treatment and prevention or as adaptogen to decrease the side effects associated with chemotherapy [33]. Previous studies reported anticancer effect of some active constituents extracted from *B. aegyptiaca*. Balanitin-6 and balanitin-7 isolated from *B. aegyptiaca* kernels showed potent anticancer effects against A549 non–small-cell lung cancer and U373 glioblastoma cell lines. In vivo study of these two steroidal saponins (Balanitin-6 and balanitin-7) increased the life span of mice bearing murine L1210 leukemia grafts to the same manner of vincristine [2]. Recent study reported the antitumor of Balanitoside isolated from *B. aegyptiaca* fruit against Ehrlich ascites carcinoma (EAC) bearing Swiss albino mice [7]. The present study was the first to provide evidence that stem bark extracts of *Balanite aegyptiaca* possess potent antiangiogenic, anti-tumor and antioxidant activities. In *ex-vivo* rat aorta ring assay, among four extracts tested, methanol extract (MBA) profoundly inhibited the sprouting of new blood vessels from explanted tissue. This inhibitory effect is more efficacious than the standard drug (Suramin). Cytotoxicity study showed that methanol is not cytotoxic towards endothelial cells (HUVEC) with IC_50_ = 72.87 μg/mL. This further supports its specific antiangiogenic action. Tumor vascularization is crucial mechanism for tumor growth and metastasis. Angiogenesis mechanism involves several steps such as endothelial cell migration which gets incorporated into the walls of growing microvessels. This process can be stimulated by growth factors such as VEGFA, placental growth factor (PIGF) and angiopoietin-1 (ANG1). Elevated levels of VEGFA expression alone are capable of triggering angiogenesis in a quiescent vasculature [34]. In this study we examined the most active extract (MBA) on the main steps of angiogenesis that is cell migration and tube formation. In addition to its ability to inhibit the expression of the main growth factor (VEGFA) that plays a fundamental role in triggering angiogenesis, was also evaluated. VEGFA is especially important to maintain permeability and integrity of blood vessels [35]. (MBA) significantly inhibited endothelial cells migration and differentiation to tube like structure at nontoxic doses on HUVEC cells. Tube formation is a major characteristic of endothelial cells in establishing new blood vessels. The Tube Formation Assay on matrigel akin the formation of microvessels in angiogenesis when the endothelial cell arranged in a three-dimensional network. VEGF is well known angiogenesis mediator in cancer and it’s the main angiogenesis growth factor that initiate different steps in the angiogenesis cascade, such as endothelial proliferation, migration and differentiation [36]. (MBA) significantly inhibited the release of VEGF from endothelial cells in a dose dependent fashion. At 50 μg/ml it inhibited VEGF expression by 31.807 % and at 100 μg/ml the suppression of VEGF was 38.76 %.

Tumor angiogenesis is critical to nurture the tumor with nutrients and oxygen. Blocking angiogenesis is a targeted approach to starve the tumor by restricting its avenues to nutrients and halting metastasis [37]. One of the primary aims of the present study was to demonstrate the effect of methanol extract (a potent antiangiogenic agent) against colon cancer propagated in nude mice to determine whether the orally administered extract was efficacious. The results exhibited that the (MBA) inhibited the tumor growth in dose-dependent manner, besides it prolonged the life span of nude mice bearing colon tumor treated with 400 mg/kg and 200 mg/kg at the end of two months. The histological study of excised tumors showed fewer blood vessels in tumors and some signs of necrosis of treated groups when compared with untreated. The antitumor activity of *B. aegyptiaca* stem bark methanol extract is thus most likely to be due to its antiangiogenic activity; moreover it showed antiproliferative effect against colon cancer cell line (HCT-116).

One of the most interesting compounds isolated from plants are alkaloids due to the their various pharmacological values, several alkaloids from botanical origin have anticancer properties and already entered the clinical arena such as taxol from (*western yew* and *Taxus brevifolia*), camptothecin from (*Camptotheca acuminate*) and homoharringtonine from (*Cephalotaxus harringtonia*) [38]. The stem bark of *B. aegyptiaca* proved to be rich in two types of alkaloids (N-trans-feruloyltyramine and N-cis-feruloyltyramine) in addition to vanillic acid, syringic acid; and 3-hydroxy-1-(4-hydroxy-3-methoxyphenyl)-1-propanone [39]. Alkaloids have considerable role in cancer treatment and prevention as mediator of signal transduction in cell proliferation as well as angiogenesis inhibitors *via* blocking Tyrosine Kinase Receptors [40]. Receptors Tyrosine Kinase Inhibitors (RTKI) such as Sunitinib, Sorafenib and Semaxanib are potent antiangiogenic alkaloids now in clinical use for cancer treatment. These alkaloids are found to exert their activity through inhibiting VEGFR. These RTK inhibitors are particularly useful in targeting cancer cells due to their dual functions in the inhibition of onco-protein signal-transduction and block the downstream angiogenesis processes [41]. In addition they often target more than one type of receptor and affect both endothelial cells and malignant cells because the receptors are expressed on both types of cell [42].

In the cytotoxicity study, the highly polar solvent extracts (methanol and water) demonstrated the highest cytotoxic activity against leukemia cancer cells (K562 and U937). Among four extracts, methanol extract exhibited most potent anti-proliferative effect on all the tested cancer cell lines (HCT-116, K562, U937 and MCF-7), noteworthy it did not exert any toxic effects on the endothelial cells. The potent antiproliferative activity of polar extracts of *B. aegyptiaca* could be speculated to high content of saponin in polar portion of stem bark of the plant [8]. Several studied reported the profound anticancer effect of saponins isolated from *B. aegyptiaca* [43].

Many studies have shown the relationship between antioxidant activity and modulation of angiogenesis [44]. The present study showed that (MBA) displayed strong DPPH scavenging ability with lowest IC_50_ (40.08 μg/ml) where it was found to contain high amounts of phenolics and flavonoids. These significant anti-angiogenic and anti-proliferative effect of methanol extract could be attributed to highly contents of anti-oxidant compounds. Research studies have shown that flavonoids as single electron donors which can stabilize and scavenge the free radicals, which in conditions of oxidative stress may initiate angiogenesis or carcinogenesis [45]. Similarly, phenolics have strong potential to interfere in a number of physiological events in biological systems, including those relating to oxidation reduction processes [46]. Furthermore phenolics and flavonoids mostly found in plants are reported to have numerous biological effects including antioxidant, anti-neovascularization, antiproliferation and anticarcinogenic properties and are therefore considered for their important dietary roles as antioxidants and chemoprotective agents. Many studies demonstrated a significant role of phenolics in growth inhibition of breast, colon, prostate, ovary, endometrium and lung cancer cells [47]. The antioxidant activity obtained from the results seems to be in a good accordance with anticancer results, moreover there is an obvious correlation between antiangiogenic and antioxidant activity, since the polyphenols inhibit the initiation and progression of angiogenesis [48]. Therefore, plant polyphenols may play an important role in halting angiogenesis and tumor progression.

## Conclusion

Stem bark of *B. aegyptiaca* methanol extract exhibit potent antiangiogenic, antiproliferative and antitumor properties, moreover it could be a promising source of anticancer agent development especially for colon, leukemia and breast cancer, and hence worthy of further investigation.

## Abbreviations

ABTS: 2,2′-azinobis(3-ethylbenzothiazoline 6-sulfonic acid
ATCC: American Type Culture Collection
CCD-18Co: Normal human colon fibroblast
CO_2_: Carbon dioxide
DMEM: Dulbecco’s Modified Eagle’s Medium
DMSO: Dimethyl Sulfoxide
DPPH: Di(phenyl)-(2,4,6-trinitrophenyl) iminoazanium
ECM: Extracellular Matrix
FRAP: Ferric reducing antioxidant power assay
HCT 116: Human colorectal carcinoma cell line K562
HIFs: Hypoxia Inducible Factors
HUVECs: Human umbilical vein endothelial cells
IC_50_: Median inhibitory concentration
MBA: Methanol extract of *Balanite aegyptiaca*
MCF-7: Human hormone sensitive and invasive breast cancer cell line
MeOH: Methyl alcohol
MTT: Thiazolyl blue tetrazolium bromide
PBS: Phosphate buffered saline
PS: Penicillin/Streptomycin
RTKI: Receptors tyrosine kinase inhibitors
TFC: Total flavonoids content
TPC: Total phenol content
VEGF: Vascular endothelial growth factor
VEGFR: Vascular endothelial growth factor receptor

## References

[1] Yadav J, Panghal M (2010). Balanites aegyptiaca (L.) Del.(Hingot): a review of its traditional uses, phytochemistry and pharmacological properties. Int J Green Pharm.

[2] Chothani DL, Vaghasiya H (2011). A review on Balanites aegyptiaca Del (desert date): phytochemical constituents, traditional uses, and pharmacological activity. Pharmacogn Rev.

[3] Shahzad A, Sahai A. "In Vitro Conservation Protocols for Some Endangered Medicinal-Plant." Recent Trends in Biotechnology and Therapeutic Applications of Medicinal Plants. Netherlands: Springer; 2013. pp. 305–321.

[4] Dinesh V (2010). Traditional uses of plants in indigenous folklore of Nizamabad District, Andhra Pradesh, India. Ethnobotanical Leaflets.

[5] Gnoula C, Mégalizzi V, De Nève N, Sauvage S, Ribaucour F, Guissou P, Duez P, Dubois J, Ingrassia L, Lefranc F (2008). Balanitin-6 and-7: diosgenyl saponins isolated from Balanites aegyptiaca Del. display significant anti-tumor activity in vitro and in vivo. Int J Oncol.

[6] Harlev E, Nevo E, Lansky EP, Lansky S, Bishayee A (2012). Anticancer attributes of desert plants: a review. Anticancer Drugs.

[7] Al-Ghannam SM, Ahmed HH, Zein N, Zahran F (2013). Antitumor activity of balanitoside extracted from Balanites aegyptiaca fruit. Journal of Applied Pharmaceutical Science.

[8] Speroni E, Cervellati R, Innocenti G, Costa S, Guerra M, Dall’Acqua S, Govoni P (2005). Anti-inflammatory, anti-nociceptive and antioxidant activities of Balanites aegyptiaca (L.) Delile. J Ethnopharmacol.

[9] Hassan L, Majid A, Iqbal M, Al Suede F, Haque R, Ismail Z, Ein O, Majid A, Ahamed MBK (2014). Crystal structure elucidation and anticancer studies of (−)-pseudosemiglabrin: a flavanone isolated from the aerial parts of tephrosia apollinea. PLoS One.

[10] Chung AS, Lee J, Ferrara N (2010). Targeting the tumour vasculature: insights from physiological angiogenesis. Nat Rev Cancer.

[11] Majid ASA, Yin ZQ, Ji D (2013). Sulphur antioxidants inhibit oxidative stress induced retinal ganglion cell death by scavenging reactive oxygen species but influence nuclear factor (erythroid-derived 2)-like 2 signalling pathway differently. Biol Pharm Bull.

[12] Höckel M, Vaupel P (2001). Tumor hypoxia: definitions and current clinical, biologic, and molecular aspects. J Natl Cancer Inst.

[13] Kowanetz M, Ferrara N (2006). Vascular endothelial growth factor signaling pathways: therapeutic perspective. Clin Cancer Res.

[14] Herbst RS (2006). Therapeutic options to target angiogenesis in human malignancies.

[15] Figg W, Folkman J. Angiogenesis: an integrative approach from science to medicine. Springer Science & Business Media; 2008.

[16] Mukhopadhyay D, Tsiokas L, Zhou X-M, Foster D, Brugge JS, Sukhatme VP (1995). Hypoxic induction of human vascular endothelial growth factor expression through c-Src activation. Nature.

[17] Hassan LEA, Koko WS, Osman E-BE, Dahab MM, Sirat HM (2011). In vitro antigiardial activity of Citrullus lanatus Var. citroides extracts and cucurbitacins isolated compounds. J Med Plants Res.

[18] Hassan LEA, Dahham SS, Fadul SM, Umar MI, Abdul Majid AS, Khaw KY, Majid AMSA. Evaluation of in vitro and in vivo anti-inflammatory effects of (−)-pseudosemiglabrin, a major phytoconstituent isolated from Tephrosia apollinea (Delile) DC. J. Ethnopharmacol. 2016. 10.1016/j.jep.2016.08.023 27553975

[19] Brown KJ, Maynes SF, Bezos A, Maguire DJ, Ford MD, Parish CR (1996). A novel in vitro assay for human angiogenesis. Lab Invest.

[20] Hassan LE, Ahamed MB, Majid AS, Baharetha H, Muslim N, Nassar Z, Majid AM (2014). Correlation of antiangiogenic, antioxidant and cytotoxic activities of some Sudanese medicinal plants with phenolic and flavonoid contents. BMC Complement Altern Med.

[21] Jost L, Kirkwood J, Whiteside T (1992). Improved short-and long-term XTT-based colorimetric cellular cytotoxicity assay for melanoma and other tumor cells. J Immunol Methods.

[22] Liang C-C, Park AY, Guan J-L (2007). In vitro scratch assay: a convenient and inexpensive method for analysis of cell migration in vitro. Nat Protoc.

[23] Nassar ZD, Aisha A, Ahamed M, Ismail Z, Abu-Salah KM, Alrokayan SA, Abdul Majid A (2011). Antiangiogenic properties of Koetjapic acid, a natural triterpene isolated from Sandoricum koetjaoe Merr. Cancer Cell Int.

[24] Chan E, Lim Y, Wong S, Lim K, Tan S, Lianto F, Yong M (2009). Effects of different drying methods on the antioxidant properties of leaves and tea of ginger species. Food Chem.

[25] Kosalec I, Bakmaz M, Pepeljnjak S, Vladimir-Knezevic S (2004). Quantitative analysis of the flavonoids in raw propolis from northern Croatia. ACTA PHARMACEUTICA-ZAGREB-.

[26] Molyneux P (2004). The use of the stable free radical diphenylpicrylhydrazyl (DPPH) for estimating antioxidant activity. Songklanakarin J Sci Technol.

[27] Andonova L, Zheleva-Dimitrova D, Georgieva M, Zlatkov A (2014). Synthesis and antioxidant activity of some 1-aryl/aralkyl piperazine derivatives with xanthine moiety at N4. Biotechnol Biotechnol Equip.

[28] Re R, Pellegrini N, Proteggente A, Pannala A, Yang M, Rice-Evans C (1999). Antioxidant activity applying an improved ABTS radical cation decolorization assay. Free Radic Biol Med..

[29] Baghel S, Shrivastava N, Baghel RS, Agrawal P, Rajput S (2012). A review of quercetin: antioxidant and anticancer properties. World J Pharm Pharmaceutical Sci.

[30] Tomayko MM, Reynolds CP (1989). Determination of subcutaneous tumor size in athymic (nude) mice. Cancer Chemother Pharmacol.

[31] Corbett T, Polin L, LoRusso P, Valeriote F, Panchapor C, Pugh S, White K, Knight J, Demchik L, Jones J. In vivo methods for screening and preclinical testing. In: Anticancer drug development guide. US: Humana Press; 2004. pp. 99–123.

[32] Cheon D-J, Orsulic S (2011). Mouse models of cancer.

[33] Ahamed M, Aisha A, Nassar Z, Siddiqui J, Ismail Z, Omari S, Parish C, Majid A (2012). Cat’s whiskers tea (Orthosiphon stamineus) extract inhibits growth of colon tumor in nude mice and angiogenesis in endothelial cells *via* suppressing VEGFR phosphorylation. Nutr Cancer.

[34] Bergers G, Benjamin LE (2003). Tumorigenesis and the angiogenic switch. Nat Rev Cancer.

[35] Keck PJ, Hauser SD, Krivi G, Sanzo K, Warren T, Feder J, Connolly DT (1989). Vascular permeability factor, an endothelial cell mitogen related to PDGF. Science.

[36] Ferrara N, Kerbel RS (2005). Angiogenesis as a therapeutic target. Nature.

[37] Dahham SS, Hassan LEA, Ahamed MBK, Majid ASA, Majid AMSA, Zulkepli NN (2016). In vivo toxicity and antitumor activity of essential oils extract from agarwood (Aquilaria crassna). BMC Complement Altern Med.

[38] Kingston D, Newman DJ (2007). Taxoids: cancer-fighting compounds from nature. Curr Opin Drug Discov Devel.

[39] Sarker S, Bartholomew B, Nash R (2000). Alkaloids from Balanites aegyptiaca. Fitoterapia.

[40] Cook KM, Figg WD (2010). Angiogenesis inhibitors: current strategies and future prospects. CA Cancer J Clin.

[41] Wilhelm SM, Adnane L, Newell P, Villanueva A, Llovet JM, Lynch M (2008). Preclinical overview of sorafenib, a multikinase inhibitor that targets both Raf and VEGF and PDGF receptor tyrosine kinase signaling. Mol Cancer Ther.

[42] Staton CA, Brown NJ, Reed MW (2009). Current status and future prospects for anti-angiogenic therapies in cancer.

[43] Ali R, Mirza Z, ASHRAF GM, Kamal MA, Ansari SA, Damanhouri GA, Abuzenadah AM, Chaudhary AG, Sheikh IA (2012). New anticancer agents: recent developments in tumor therapy. Anticancer Res.

[44] Jackson J, Higo T, Hunter W, Burt H (2006). The antioxidants curcumin and quercetin inhibit inflammatory processes associated with arthritis. Inflamm Res.

[45] Nijveldt RJ, Van Nood E, Van Hoorn DE, Boelens PG, Van Norren K, Van Leeuwen PA (2001). Flavonoids: a review of probable mechanisms of action and potential applications. Am J Clin Nutr.

[46] Balasundram N, Sundram K, Samman S (2006). Phenolic compounds in plants and agri-industrial by-products: antioxidant activity, occurrence, and potential uses. Food Chem.

[47] Galati G, O’Brien P (2004). Potential toxicity of flavonoids and other dietary phenolics: significance for their chemopreventive and anticancer properties. Free Radic Biol Med.

[48] Walter A, Etienne-Selloum N, Brasse D, Schleiffer R, Bekaert V, Vanhoutte P, Beretz A, Schini-Kerth V (2009). Red wine polyphenols prevent acceleration of neovascularization by angiotensin II in the ischemic rat hindlimb. J Pharmacol Exp Ther.

